# High-resolution source inversion of 2024 Noto Peninsula earthquake tsunami with modeling error corrections

**DOI:** 10.1038/s41598-025-08978-0

**Published:** 2025-07-10

**Authors:** Tomohiro Takagawa, Yu Chida, Takashi Fujiki, Koji Kawaguchi

**Affiliations:** 1 Port and Airport Research Institute, Yokosuka, Japan; 2 Tsunami and Storm Surge Research Group, Port and Airport Research Institute, National Institute of Maritime, Port and Aviation Technology, 1-1-3, Nagase, Yokosuka, 239-0826 Japan; 3 National Institute for Land and Infrastructure Management, Yokosuka, Japan

**Keywords:** Tsunami, Waveform inversion, Modeling error, Adjoint synthesis, Inundation height, 2024 Noto Peninsula earthquake, Physical oceanography, Natural hazards, Ocean sciences, Solid Earth sciences

## Abstract

On New Year’s Day, January 1, 2024, a significant earthquake struck the Noto Peninsula, Japan, triggering a tsunami with a maximum height of exceeding 5 m along the coast. This rare and destructive event is associated with several active submarine faults in the area, which are inferred to have ruptured simultaneously. However, due to the lack of direct observation from the ocean, the precise nature of the initial disturbance remains uncertain. In this study, we utilized the latest high-resolution adjoint synthesis inversion technique to analyze the tsunami’s initial state based on observed water level and velocity fluctuations. Furthermore, we propose a method to correct modeling errors in amplitude and phase, thereby reducing misfits in trace heights: the geometric mean $K$ improves from 1.16 to 0.99, and the geometric standard deviation $\kappa$ decreases from 1.34 to 1.29. Our estimated tsunami source model identified three distinct uplift peaks, each approximately 3 m in height, in the offshore region. The model accurately reproduced both offshore and onshore observation data. This research provides a robust framework for understanding tsunami generation mechanisms and improving hazard assessment models. By offering accurate initial conditions for tsunami simulations, our findings contribute to better disaster preparedness and mitigation strategies, particularly for coastal regions prone to similar events.

**Supplementary Information:**

The online version contains supplementary material available at 10.1038/s41598-025-08978-0.

## Introduction

**Fig. 1:**
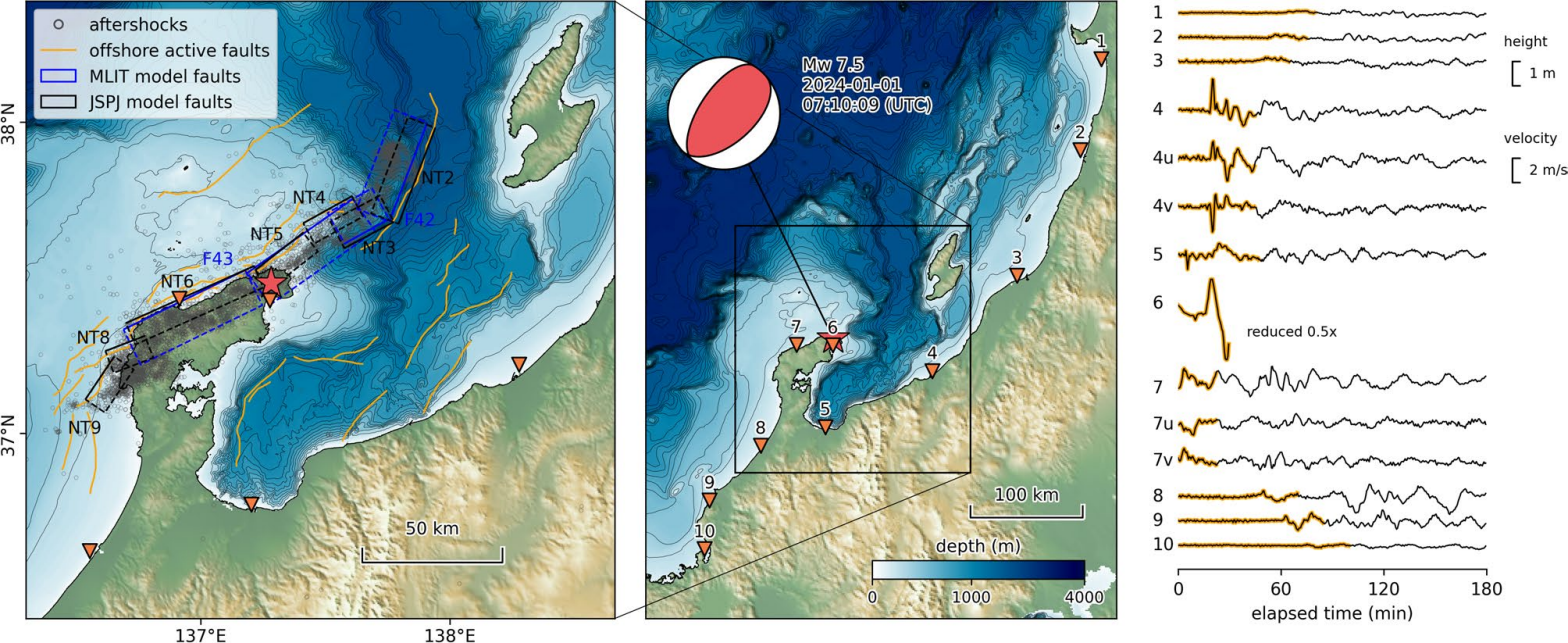
Active submarine faults around the Noto Peninsula (left), tsunami observation stations (center), and observed waveforms (right). Waveforms labeled with “u” or “v” after the station number represent eastward and northward velocities, respectively, while those without letters correspond to observed water levels. Short-period wave and tidal components have been removed. The thick orange line indicates the data section used for waveform inversion. The station names are as follows: 1: Akita, 2: Sakata, 3: Niigata, 4: Naoetsu, 5: Toyama, 6: Iida, 7: Wajima, 8: Kanazawa, 9: Fukui, 10: Tsuruga. The maps and plots were created using Matplotlib version 3.10.1 (https://matplotlib.org/) and ObsPy version 1.4.1 (https://github.com/obspy/obspy).

On January 1, 2024, a magnitude 7.5 earthquake^1^ struck the Noto Peninsula in Japan, producing strong tremors with a maximum intensity of 7, the highest level on the Japan Seismic Intensity Scale^2^. The earthquake is hypothesized to have resulted from the simultaneous rupture of multiple active submarine faults extending from the ocean to the northern edge of the Noto Peninsula^3,4^. Interferometric SAR analysis revealed a maximum elevation change of up to 4 m in the northern portion of the Noto Peninsula^5^, along with the transformation of shallow seabed areas into land^6^. Evidence of seabed displacement and submarine landslides was further elucidated through seafloor topography surveys^7,8^. These disturbances to the seafloor generated a tsunami, which subsequently caused extensive flooding damage. The initial analysis of satellite images^9^ and subsequent on-site surveys^10,11^ documented the spatial distribution and heights of the tsunami as it impacted land. Notably, the tsunami trace heights exceeded 5 m near Iida Port on the eastern coast of the Noto Peninsula and Naoetsu Port in Niigata Prefecture, located on the opposite coast (Fig. 1). Additionally, at Toyama Port, significant water level fluctuations were observed earlier than expected based on the estimated tsunami travel time from its source. This anomaly is attributed to multiple submarine landslides in the bay, which generated another tsunami^12^.

This study focuses on the water surface disturbance off the northeastern coast of Noto Peninsula, which is considered the primary cause of the large-scale flooding with heights exceeding 5 m. Because this is a marine area lacking a seafloor observation network, it is necessary to estimate the changes that occurred during the earthquake using data from surrounding regions. The analysis of tsunami waveforms provides an effective approach for investigating seafloor changes in areas distant from earthquake and GNSS observation networks^13^. In this region, the distribution of active submarine faults has been identified through seafloor topography and seismic surveys^14^. These findings have resulted in the proposal of several rectangular fault models, which are utilized in tsunami risk assessments^15,16^ (Fig. 1).

Efforts have been made to estimate tsunami sources based on these fault models. Fujii & Satake^17^ applied the JSPJ fault model (Fig. 1) in a joint inversion analysis using tsunami waveform data and GNSS data. Their results indicated a significant slip on subfault NT4, while little to no slip was estimated on subfaults NT2 and NT3. Masuda et al.^18^ highlighted that Fujii & Satake^17^ did not account for the response characteristics of tide stations, which measure water levels in wells connected to the sea through narrow pipes. They demonstrated that the MLIT fault model accurately reproduced the inundation area on the east coast of the Noto Peninsula near Iida when ruptures occurred on faults F43 and the western portion of F42 (Fig. 1). Yamanaka et al.^19^ used time-series water level data extracted from video footage at Iida Port to estimate the initial water level distribution off the northeastern coast of the Noto Peninsula. Their study primarily aimed to reproduce waveforms in this region to analyze tsunami amplification characteristics in Iida Bay and was not intended to model tsunamis propagating to other areas.

This study differs from previous research by directly estimating the initial water level distribution of the tsunami through the analysis of observational data, including both water levels and flow velocities, without assuming any fault. A high-resolution inversion analysis with a spatial resolution of 1 km was performed using the adjoint synthesis method proposed by Takagawa et al.^20^. Additionally, we developed a methodology for correcting modeling inaccuracies in theoretical waveforms, categorizing these issues as either phase or amplitude errors. Phase errors in theoretical waveforms indicate strong temporal correlations in the time-series data, which can negatively impact inversion results. Spatial regularization is commonly introduced to prevent analysis degradation due to overfitting to data noise^21^. However, such regularization often suppresses the amplitude of the initial source, leading to underestimation of tsunami wave heights and inundation on land. To address this, we propose a method for correcting both phase and amplitude errors in theoretical waveforms. This new correction method will be applied to analyze the Noto Peninsula earthquake tsunami, verifying its effectiveness and clarifying the actual initial water level disturbance that caused the destructive tsunami.

## Methods

The initial water level distribution was estimated using the regularized least squares method (e.g.,^21^). The distribution is represented as a linear combination of unit sources. The propagation of the tsunami generated by each unit source is simulated, and the resulting waveform at each observation point, referred to the Green’s function, is stored in a database. The theoretical waveform is then synthesized as a linear combination of these Green’s functions^22,23^, as shown below:

1 $$\begin{array}{c}\mathbf{y}=\mathbf{Ax}\end{array}$$

where $\mathbf{x}$ is a vector of coefficients of the linear combination of the unit sources, the matrix $\mathbf{A}$ contains the Green’s functions for each observation point, and $\mathbf{y}$ is the theoretical waveform. The square error between the observed and theoretical waveforms is minimized to estimate $\mathbf{x}$. However, in the cases of underdetermined problems with relatively little data, a unique solution cannot be obtained. Consequently, $\mathbf{x}$ is estimated by minimizing the following function $f$, which incorporates a regularization term.

2 $$\begin{array}{c}f\left(\mathbf{x}\right)={\left|{\mathbf{y}}_{\mathbf{o}\mathbf{b}\mathbf{s}}-\mathbf{A}\mathbf{x}\right|}^{2}+{\gamma }^{2}{\left|{\varvec{\Delta}}\mathbf{x}\right|}^{2}\end{array}$$

where the second term on the right-hand side represents the regularization term. ${\varvec{\Delta}}$ is a Laplace operator used to calculate the smoothness of the source^20^, and ${\gamma }^{2}$ is a hyperparameter that controls the strength of the regularization. The resolution of the inversion analysis can be enhanced by reducing the spatial extent of the unit source of the Green’s function and positioning it more densely. However, the achievable resolution is practically limited because the number of simulations required to compute the Green’s function increases proportionally to the square of the resolution. For example, if the resolution is increased tenfold, the number of simulations increases by a factor of 100.

Adjoint inversion^24–26^ enables high-resolution analysis with a computational load of only a few dozen simulations by efficiently calculating the gradient during iterative optimization of Eq. 2 using adjoint operations. This approach avoids the explicit calculation of the elements of matrix $\mathbf{A}$, which is computationally expensive. However, Takagawa et al.^20^ demonstrated that by employing an adjoin operator, the elements of matrix $\mathbf{A}$ can be efficiently calculated, even for a high-density Green’s function database. This method eliminates the need for simulations during the iterative optimization of Eq. 2. The fluctuation observed at point ${\xi }_{o}$ at time $t$ caused by a unit disturbance at point ${\xi }_{s}$ at time $0$, i.e. the Green’s function $G\left(t\right)$, can be calculated in two distinct ways as follows:

3 $$\begin{array}{*{20}c} {G\left( t \right) = \langle {\mathcal{L}}\xi_{s} ,\xi_{o} \rangle = \langle \xi_{s} ,{\mathcal{L}}^{\dag } \xi_{o} \rangle } \\ \end{array}$$

where ${\xi }_{s}$ is the source function and ${\xi }_{o}$ is the observation function. The operator $\mathcal{L}$ is a linear operator that represents the propagation of a tsunami from time $0$ to time $t$, ${\mathcal{L}}^{\dag }$ is the adjoint operator of $\mathcal{L}$, and $\left\langle { \cdot , \cdot } \right\rangle$ represents the inner product. Equation 3 applies, even for non-point sources and non-point observations. The Green’s function of a tsunami propagating over complex bathymetry is typically calculated using a discretized numerical model. This corresponds to the middle expression in Eq. 3, where $\mathcal{L}$ represents the numerical simulation. To compute the Green’s function for various tsunami sources using this method, simulations must be performed for the number of tsunami sources as initial conditions for each ${\xi }_{s}$. Alternatively, when Green’s function is calculated using the right-hand side of Eq. 3, an adjoint simulation is performed with the observation function ${\xi }_{o}$ as the initial condition. By taking the inner product of the resulting wave field (referred to as the adjoint state) with different tsunami sources, it becomes possible to compute waveforms for multiple initial conditions without repeating numerical simulations. For computations using the operator $\mathcal{L}$, the computational load on the Green’s function database is proportional to the number of sources, whereas for calculations using the adjoint operator ${\mathcal{L}}^{\dag }$, it is proportional to the number of observation functions. When a large number of small unit sources are arranged in a high-density pattern for high-resolution analysis, the latter approach, where computational effort does not increase with the number of sources, is more advantageous. In this study, the adjoint operation of ${\mathcal{L}}^{\dag }$ was computed with the adjoint tsunami model developed by Takagawa et al.^20^.

The tsunami waveform data from the Noto Peninsula Earthquake includes observations from the area where the tsunami originated. To mitigate the effects of vertical displacement at the observation facilities, we performed the inversion using the time-derivative waveform proposed by Kubota et al.^27^. The objective function to be minimized is as follows:

$$f\left(\mathbf{x}\right)={\left|\frac{d{\mathbf{y}}_{\mathbf{o}\mathbf{b}\mathbf{s}}}{dt}-\frac{\mathbf{A}}{dt}\mathbf{x}\right|}^{2}+{\gamma }^{2}{\left|{\varvec{\Delta}}\mathbf{x}\right|}^{2}$$

$$= \left| {\left[ {\begin{array}{*{20}c} {\displaystyle \frac{{d{\mathbf{y}}_{{{\mathbf{obs}}}} }}{dt}} \\ 0 \\ \end{array} } \right] - \left[ {\begin{array}{*{20}c} {\displaystyle \frac{{d{\mathbf{A}}}}{dt}} \\ {\gamma^{2} {{\varvec{\Delta}}}} \\ \end{array} } \right]{\mathbf{x}}} \right|^{2} = \left| {{\mathbf{y^{\prime}}}_{{{\mathbf{obs}}}} - {\mathbf{A^{\prime}x}}} \right|^{2}$$

4 $$f\left(\mathbf{x}\right)={\left|\frac{d{\mathbf{y}}_{\mathbf{o}\mathbf{b}\mathbf{s}}}{dt}-\frac{\mathbf{dA}}{dt}\mathbf{x}\right|}^{2}+{\gamma }^{2}{\left|{\varvec{\Delta}}\mathbf{x}\right|}^{2}$$

, where

$${\mathbf{y}}^{\prime }_{{{\mathbf{obs}}}} = \left[ {\begin{array}{*{20}c} {\displaystyle \frac{{d{\mathbf{y}}_{{{\mathbf{obs}}}} }}{dt}} \\ 0 \\ \end{array} } \right],{ }{\mathbf{A}}^{\prime } = \left[ {\begin{array}{*{20}c} {\displaystyle \frac{{d{\mathbf{A}}}}{dt}} \\ {\gamma^{2} {{\varvec{\Delta}}}} \\ \end{array} } \right].$$

Minimizing this objective function $f$ is equivalent to finding $\mathbf{x}$ such that the gradient of $f$ is zero. The estimated value of $\widehat{\mathbf{x}}$ was obtained by solving the following equation using the conjugate gradient method.

5 $$\begin{array}{*{20}c} {\displaystyle \frac{1}{2}\frac{\partial f}{{\partial {\mathbf{x}}}} = {\mathbf{A}}^{\prime \top } {\mathbf{A^{\prime}\widehat{x}}} - {\mathbf{A}}^{\prime \top } {\mathbf{y}}_{{{\mathbf{obs}}}}^{\prime } = 0} \\ \end{array}$$

The Green’s function used in the inversion represents an approximation of natural phenomena. Consequently, the results are subject to various modeling errors^28^, including those arising from governing equations, numerical discretization, and boundary conditions, such as water depth. For example, it has been pointed out that when linear equations are used, amplitudes become overestimated because energy dissipation due to bottom friction is not taken into account^29^, and that neglecting dispersion causes the phase to advance more rapidly than in reality, thereby affecting source inversion^30^. Additionally, errors in the tsunami generation time can affect the results of the inverse analysis. To address these modeling errors, two parameters are introduced in this study to adjust Green’s function. The first is the amplitude correction parameter, denoted as ${\alpha }_{k}$, and the second is the phase correction parameter, denoted as ${\tau }_{k}$. $k$ represents the index of the observation point, and these parameters are defined for each observation point. The Green’s function, incorporating these corrections, is expressed as follows:

6 $$\begin{array}{c}{\alpha }_{k}G\left(t-{\tau }_{k}\right).\end{array}$$

Phase correction has been employed in previous tsunami source analyses, and its effectiveness has been demonstrated^20,30^. However, a unified method for phase correction has not yet been established. Separation of errors into amplitude and phase components has been applied to performance evaluation of observation systems and to the optimization of observation-station layouts^31^. The motivation for introducing amplitude correction is to address modeling errors in amplitude, and to prevent the underestimation of amplitude caused by the regularization term in inversion analysis. The amplitude of a tsunami is a critical factor in evaluating the forces exerted on structures and estimating inundation damage. Therefore, a tsunami source model that produces amplitudes closely matching actual measurements in coastal areas is essential for quantitatively understanding the relationship between external forces and resulting damage in coastal regions and on land.

As outlined earlier, the optimization problem under consideration requires the estimation of three distinct types of hyperparameters: regularization, phase correction, and amplitude correction. The regularization hyperparameter, ${\gamma }^{2}$, is determined via Cross Validation (CV) under the condition of ${\alpha }_{k}=1$ and ${\tau }_{k}=0$. The training data used for inversion corresponds to the section shown in Fig. 1, while the test data for CV includes this section along with data from the following 60 minutes^32^. After fixing the regularization parameter ${\gamma }^{2}$, the phase correction parameter ${\tau }_{k}$ for each observation point is iteratively estimated through another CV. First, an observation point is selected, and tsunami source inversion is performed excluding the data observed at that point. Second, the waveform of the excluded point is computed from the estimated tsunami source. Third, the estimated waveform at the excluded station is shifted in the time direction, and the amount of shift that minimizes the squared error with the observed waveform serves as the temporal estimate of the phase correction parameter ${\tau }_{k}$. The selection of observation points and updates to the phase correction parameters are repeated until changes in the parameters become sufficiently small. When the observation points used for the inversion are changed, the estimated source parameters are affected because error factors, such as the tsunami’s propagation distance from the source and the complexity of the surrounding bathymetry, differ^33^. Using the aforementioned algorithm, however, one obtains a set of optimal ${\tau }_{k}$ values that change very little even when the excluded observation points are varied. In other words, overfitting to specific station data or error characteristics is suppressed, yielding robust estimates. Here, the optimal shift is estimated by grid search. The grid interval must be significantly smaller than the observed tsunami period of about 10 min. However, since an excessively small interval would increase computational load, a 5-s interval was chosen. The search range was set to 5 min—half of the target tsunami period—to avoid cycle skipping. The relationship between time shifts and misfits for each observation point is shown in Supplementary Fig. S1.

Amplitude correction coefficients ${\alpha }_{k}$ for each observation point were estimated by iteratively updating the coefficients using the following equation, which represents the ratio of the estimated waveform amplitude to the observed waveform amplitude, until the values converged:

7 $$\begin{array}{c}{\alpha }^{n+1} _{k}=\displaystyle \frac{{a}_{est}}{{a}_{obs}}{\alpha }^n_{k}\end{array}$$

where ${a}_{obs}$ and ${a}_{est}$ are the amplitudes of the observed and estimated waveforms, respectively. Since it is unrealistic for the correction coefficients to deviate significantly from 1, they are constrained to a range of ${{p}_{amp}}^{-1}$ to ${p}_{amp}$ . ${p}_{amp}$ is a parameter that constrains the range of ${\alpha }_{k}$ and satisfies ${p}_{amp}\ge 1$. The case ${p}_{amp}=1$ is equivalent to not applying any correction for amplitude-modeling errors. Amplitude is defined as the difference between the maximum and minimum values within the inversion section. If the amplitude of the estimated waveform is smaller than the observed amplitude, the Green’s function is adjusted to be smaller due to the amplitude correction. Consequently, the deviation of the tsunami source contributing to that observation is estimated to be larger in the inversion. This parameter update was repeated until the tsunami source estimation results converged. Finally, the estimated tsunami source was used to perform tsunami propagation and inundation simulations based on the nonlinear long-wave Eq. ^34^, yielding estimates of the final waveforms at observation points and the inundation/runup heights on land.

## Data

The primary targets for tsunami source inversion are the observation data (Fig. 1) from Coastal Wave Gauges (CWGs) of the Nationwide Ocean Wave information network for Ports and HArbourS (NOWPHAS) and water level change data extracted from video footage taken at Iida Port^19^. At CWGs, water levels and flow velocities are measured using ultrasound. We used water level data from 9 locations (Akita, Sakata, Niigata, Naoetsu, Toyama, Wajima, Kanazawa, Fukui, and Tsuruga) and eastward and northward flow velocity data from 2 locations near the source (Naoetsu and Wajima). The original data acquired were between December 25, 2023 and January 4, 2024, with a sampling interval of 0.5 s. A one-minute moving average was applied to remove short-period components related to wind waves, and the four major tidal components (M_2_, S_2_, K_1_, and O_1_) were determined through the least squares method^35^ and subsequently removed. Since wind waves have a period of approximately 10 s, the moving-average window size was set to one minute to attenuate these components. The processed data were then down sampled to 30-s intervals and used in the inversion analysis.

The extent of the initial water level disturbance was constrained using the distribution of aftershocks recorded within 30 days of the main shock^36^. Vertical displacement data on land, observed by GNSS stations^37^ and estimated through Interferometric Synthetic Aperture Radar (InSAR) analysis of ALOS2^5^, were also incorporated into the inversion. The weight of the land data, ${w}_{land}$ , was set to 0.1 times that of the tsunami observation data based on preliminary investigations. In these preliminary investigations, we found that changing the weight of the land data altered the absolute heights on land and offshore relative to one another, yet had almost no effect on the positions of the resulting peaks (Supplementary Fig. S2). The variable controlling the range of the amplitude-correction coefficient, ${p}_{amp}$, was set to 2; although larger values of ${p}_{amp}$ relax the constraint, values above 2 had only a limited impact on the estimated source (Supplementary Fig. S2). Bathymetric data for adjoint simulations to construct the Green’s function database were derived from 15-arc-second GEBCO grid data^38^, with coastal areas corrected using M7000 datasets^39^. For the tsunami inundation simulation based on the nonlinear long-wave equation, topographic data created by MLIT^16^ were used. This data employs nesting grids where the grid size gradually decreases in a stepwise manner from offshore to coastal and inland areas, with a minimum grid size of 50 m.

The tsunami trace height dataset from the post-event survey^11^ was used for tsunami source validation. This dataset was compiled in accordance with the post-tsunami survey field guide^40^. The dataset includes annotations providing information such as the classification of tsunami height (e.g., run-up height or inundation height), the influence of splash or wind/swell waves, and a reliability level ranging from A to D. Only data with a reliability level of A or B were used in this study. Preliminary investigations (see Supplementary Fig. S3) revealed a tendency for trace heights to be higher at open coasts facing northwest, which are directly exposed to seasonal northwest waves in this area^41^ . Conversely, trace heights tend to be lower at sites protected by small-scale breakwaters that were not represented in the 50 m mesh simulations (Supplementary Fig. S3). Accordingly, data influenced by wave action, splash effects, and the presence of protective structures were excluded from the subsequent validation process.

## Results

**Fig. 2:**
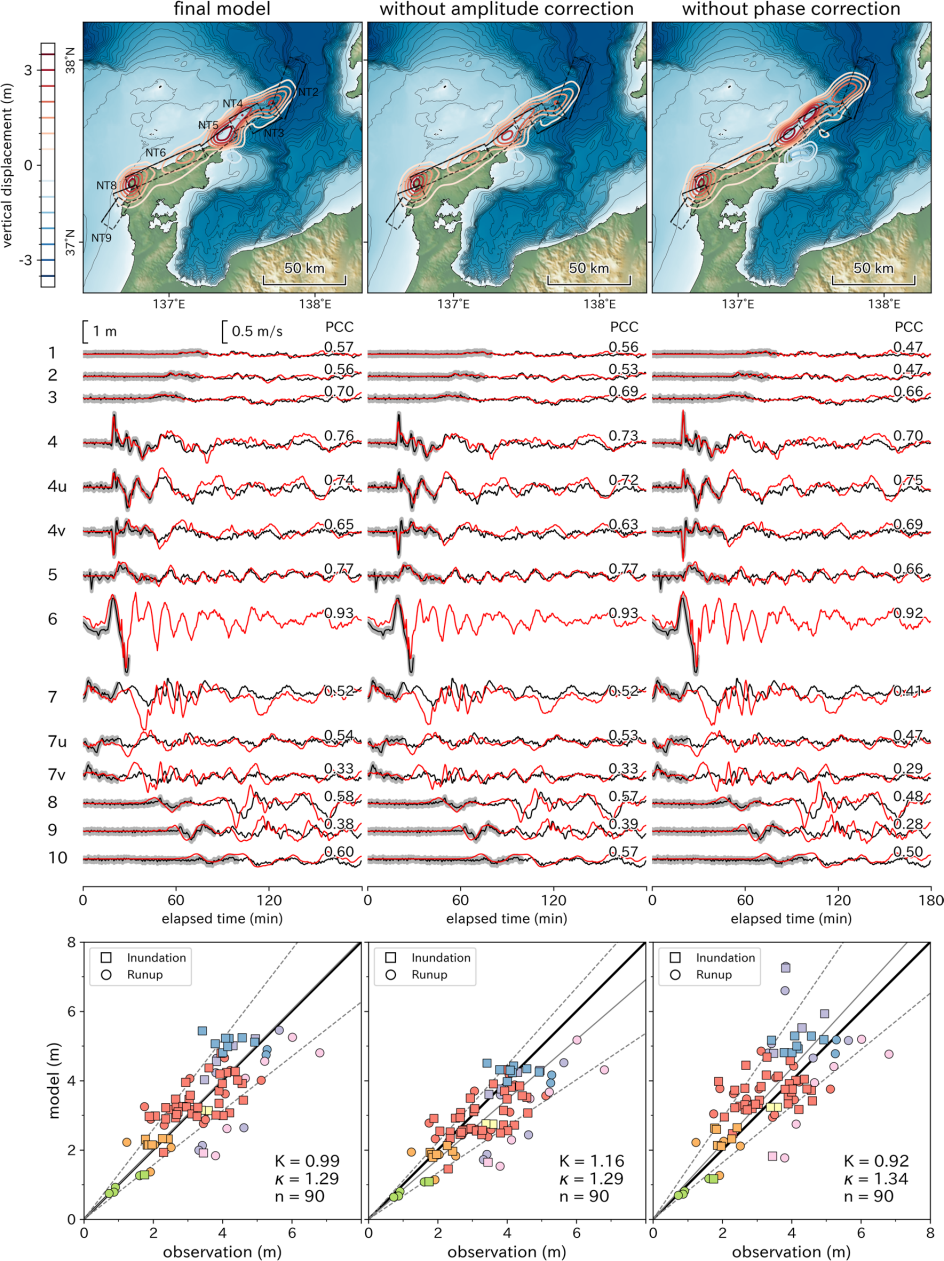
Estimation of initial water level distribution with and without correction (top), corresponding water level and flow velocity waveforms (middle), and inundation/runup heights (bottom). The left panels show results with both amplitude and phase corrections, the center panels show results without amplitude correction, and the right panels show results without phase correction. Observed waveforms are shown as black lines, and estimated waveforms are shown as red lines. Thick gray lines indicate the sections used for the inversion. Pearson Correlation Coefficients (PCCs) between observed and estimated values are shown on the right-hand side of each waveform. Marker colors in the bottom panels correspond to measurement areas shown in Supplementary Fig. S3. Aida^42^'s geometric mean $K$, geometric standard deviation $\kappa$, and the total number of data points $n$ are indicated in each plot. In the bottom panels, the thick solid line is the 1:1 line (slope of 1 through the origin), the thin solid line has a slope of $1/K$, and the thin dashed lines indicate the bounds of one geometric standard deviation with slopes of $1/(\kappa K)$ and $\kappa /K$. The maps and plots were created using Matplotlib version 3.10.1 (https://matplotlib.org/).

The final tsunami source model, corrected for modeling errors in phase and amplitude, is shown in Fig. 2. Off the northeastern coast of the Noto Peninsula, uplift peaks of up to 3.3 m and 3.0 m were estimated at locations corresponding to NT5 and NT4, respectively. Another uplift peak of up to 2.6 m was estimated near the boundary between NT3 and NT2. This location corresponds to a region with a characteristic steep topography, where contour lines bend in a northwesterly direction (Supplementary Fig. S3). On land, two uplift peaks were identified: one of 3.3 m in the westernmost part of NT6 and another of 1.8 m in the eastern part. The Pearson Correlation Coefficients (PCCs) between the observed and estimated waveforms are shown in the middle panels of Fig. 2. PCC values range from –1 to + 1, with values closer to 1 indicating better agreement between observed and estimated waveforms. Positive correlations were observed at all points. The waveforms calculated for each observation point using the nonlinear long-wave model generally reproduced the characteristics of the observed waveforms. The observed and estimated trace heights also showed good agreement, with Aida^42^’s geometric mean $K$ of 0.99 and geometric standard deviation $\kappa$ of 1.29, as shown in the bottom panel of Fig. 2. In the final model, the ratio of observed to model trace heights is close to unity; however, in the lower panel of Fig. 2, the blue markers show a slight tendency toward overestimation. This area—known as Shiromaru—experienced locally elevated tsunami heights and extensive inundation damage^10^. Shiromaru is a small, isolated bay with a narrow mouth of roughly 300 m, and a 50 m grid spacing in the model likely provided insufficient resolution, which may have introduced this bias. Validation using higher-resolution bathymetric data remains a task for future work.

To evaluate the effects of the proposed corrections, Fig. 2 also presents the estimation results without amplitude correction and without phase correction. When the amplitude correction is omitted, the distribution of the uplift area of the source changes minimally; however, the amount of uplift off the northeastern coast of the Noto Peninsula is estimated to be slightly smaller. Without amplitude correction, the PCCs at observation points 1 to 4 decreased slightly. Notably, the amplitude of the characteristic first wave of the water level and flow velocity at Naoetsu (No. 4) showed a significant decrease. Consequently, the geometric mean of the trace heights was 1.16, indicating an underestimation of about 16%.

**Fig. 3:**
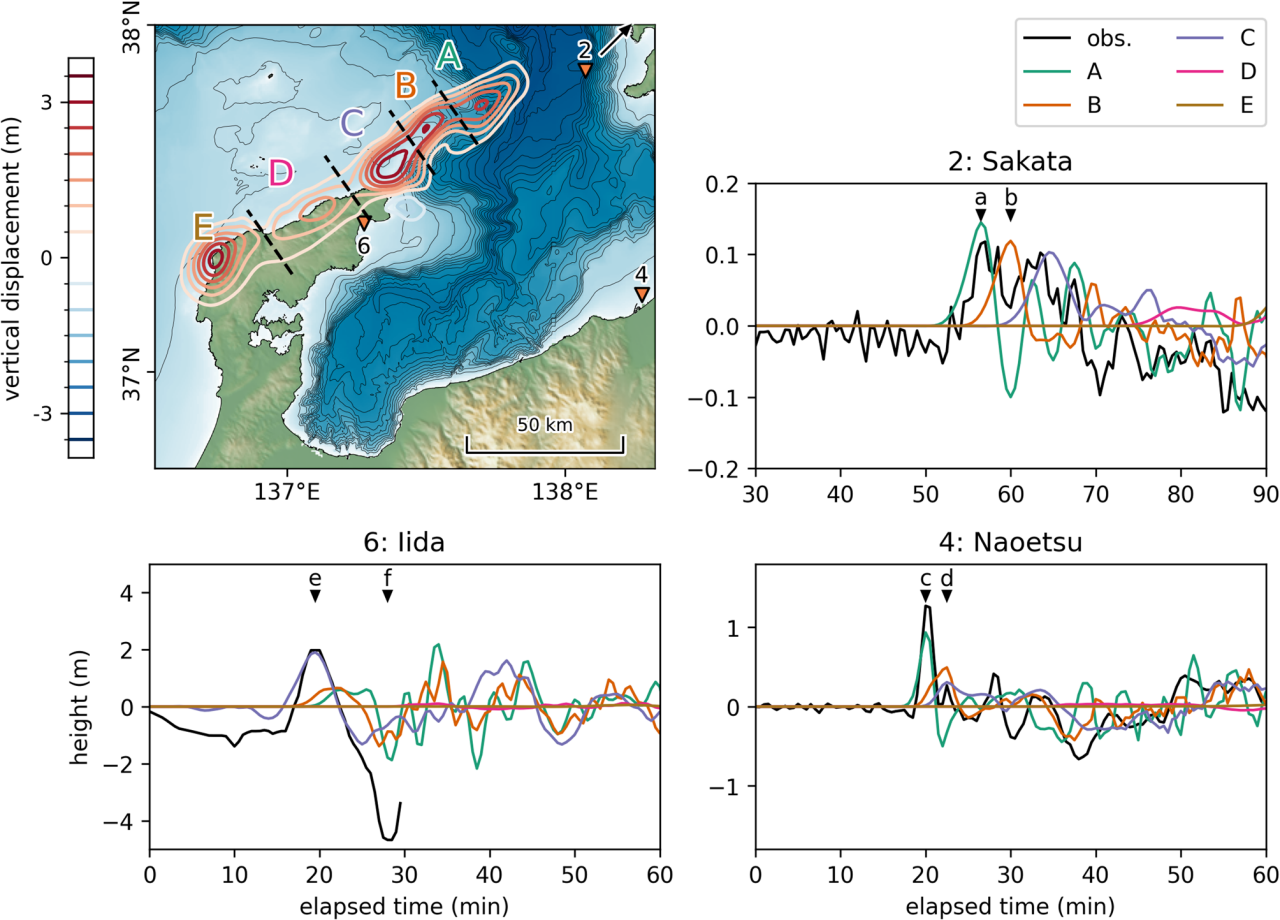
Segmentation of the initial water level distribution and comparison of the tsunami waveform generated for each segment with the observed waveforms. Observation station No. 2, Sakata, lies outside the bounds of the map. Its direction is indicated by a black arrow, and the exact location of Sakata can be found in Fig. 1. The map and plots were created using Matplotlib version 3.10.1 (https://matplotlib.org/).

When the phase correction is omitted, the uplift peaks off the northeastern coast of the Noto Peninsula are estimated to shift by 6 km to the NNE, deviating from the distribution of active submarine faults. The three offshore uplift peaks were estimated to be higher, and the PCCs of most waveforms decreased. The geometric mean of the trace heights was 0.92, indicating an overestimation of about 8%. The standard deviation was larger than that obtained with corrections, reflecting greater variability in the results. Given the five distinct peaks in the estimated tsunami source, the area was divided into segments as shown in Fig. 3, and the tsunami waveforms generated for each segment were compared with the observed waveforms. Note that in the figure, the segments are labeled with uppercase letters A–E, while the points in the time-series waveforms are represented by lowercase letters a–f. The first peak of the observed waveform at Sakata (point a in the figure), located far to the northeast of the source, is attributed to the uplift source in segment A. The peak corresponding to the uplift source in segment B is not visible in the observed waveform (point b) because it is superimposed on the backwash generated by the source in segment A.

A distinctive first peak (point c) was observed at Naoetsu, almost certainly caused by the uplift source in segment A. The waveform from segment A then transitions into a backwash (point d), but here a small push wave is observed. This results from the superposition of push waves from the uplift sources of segments B and C. The impact of the tsunami sources in segments D and E is minimal. The first wave of the observed waveform at Iida Port, with a peak height of about 2 m (point e), is almost entirely reproduced by the uplift source in segment C. The overlapping of the three backwashes from segments A, B, and C is likely responsible for the subsequent large backwash with a deviation of −5 m (point f).

## Conclusions

The initial water level distribution of the Noto Peninsula earthquake tsunami was estimated using observed tsunami waveforms and land displacement data. This study is notable for directly estimating the water level distribution without relying on a fault model. Furthermore, it stands out from previous research through its utilization of adjoint synthesis to enable high-resolution analysis at a spatial resolution of 1 km. The results of a tsunami source inversion provide insights into the changes that occurred on the seafloor off the northeastern coast of the Noto Peninsula. The following represents a concise overview of the principal findings:

- A method for correcting modeling errors in amplitude and phase has been proposed. When applied, this correction allows the tsunami source to be estimated in a position that closely corresponds to active submarine faults, improving the accuracy of both tsunami waveforms and trace height estimates.
- The estimated initial water level distribution model reveals the presence of uplift peaks at the locations of the active submarine faults NT5 and NT4, with heights of 3.3 m and 3.0 m, respectively. Additionally, an uplift peak of 2.6 m is estimated near the boundary between NT2 and NT3.
- The distinctive first peak wave that struck Naoetsu is attributed to the uplift peak estimated near the boundary between NT2 and NT3.
- The first peak wave that struck Iida is attributed to the uplift peak at the NT5 location.
- To reproduce the waveforms following the first peak waves observed at Naoetsu and Iida, it was necessary to add an uplift peak at the NT4 location.
- The proposed initial water level distribution is notable for its ability to accurately reproduce both the observed offshore waveforms and the measured trace heights on land. This provides a practical initial tsunami condition for quantifying the height and force of the tsunami that impacted damaged protective structures and buildings.

By providing precise initial conditions for tsunami simulations, our findings enhance disaster preparedness and mitigation efforts, especially in coastal areas vulnerable to similar events.

## Electronic supplementary material

Below is the link to the electronic supplementary material.

Supplementary Material 1

Supplementary Material 2

## Data Availability

The digital data for the final tsunami source model is provided as Supplementary Table S1. The software used for tsunami simulation is available from https://github.com/tomographyyy/tandem. Bathymetric data are available from http://dx.doi.org/10.5285/f98b053b-0cbc-6c23-e053-6c86abc0af7b. Other data are available from the corresponding author upon reasonable request.
