# Correction: High-resolution source inversion of 2024 Noto Peninsula earthquake tsunami with modeling error corrections

**DOI:** 10.1038/s41598-025-23121-9

**Published:** 2025-10-15

**Authors:** Tomohiro Takagawa, Yu Chida, Takashi Fujiki, Koji Kawaguchi

**Affiliations:** 1 Port and Airport Research Institute, Yokosuka, Japan; 2 Tsunami and Storm Surge Research Group, Port and Airport Research Institute, National Institute of Maritime, Port and Aviation Technology, 1-1-3, Nagase, Yokosuka, 239-0826 Japan; 3 National Institute for Land and Infrastructure Management, Yokosuka, Japan

Correction: *Scientific Reports* 10.1038/s41598-025-08978-0, published online 10 July 2025

The original version of this Article contained typesetting errors in Equations  4 and 7.

The incorrect and correct Equations appear below.

Incorrect Equation 4

$$f\left(\text{x}\right)={\left|\frac{d{\text{y}}_{\text{obs}}}{dt}-\frac{\text{A}}{dt}\text{x}\right|}^{2}+{\gamma }^{2}{\left|{\Delta {\rm x}}\right|}^{2}$$

Correct Equation 4

$$f\left(\text{x}\right)={\left|\frac{d{\text{y}}_{\text{obs}}}{dt}-\frac{d\text{A}}{dt}\text{x}\right|}^{2}+{\gamma }^{2}{\left|{\Delta {\rm x}}\right|}^{2}$$

Incorrect Equation 7

$${\alpha }_{k}=\frac{{a}_{est}}{{a}_{obs}}$$

Correct Equation 7

$${\alpha }_{k}^{n+1}=\frac{{a}_{est}}{{a}_{obs}}{\alpha }_{k}^{n}$$

The original Article has been corrected.

