# Supplementary material for: High-resolution source inversion of 2024 Noto Peninsula earthquake tsunami with modeling error corrections
Source: Sci Rep. 2025 Jul 10;15:24889. doi: 10.1038/s41598-025-08978-0 (PMC12246216; doi:10.1038/s41598-025-08978-0)
Supplement: Supplementary file 2 — Supplementary Material 2 [file 41598_2025_8978_MOESM2_ESM.docx]

Supplementary Information for

**High-resolution Source Inversion of 2024 Noto Peninsula Earthquake Tsunami with Modeling Error Corrections**

Tomohiro Takagawa^1, *^, Yu Chida^2^, Takashi Fujiki^1^, and Koji Kawaguchi^1^

^1^Port and Airport Research Institute, Yokosuka, Japan

^2^National Institute for Land and Infrastructure Management

*takagawa-t@p.mpat.go.jp

**Contents of this file**

Figures S1, S2 and S3

**Additional Supplementary Information (File uploaded separately)**

Table S1

**Introduction**

The relationship between time shifts and misfits for each observation point is shown in Fig. S1. The results of preliminary investigations into the influence of model parameters on tsunami source inversion and the effects of wind waves and structural protection on tsunami trace height measurements are summarized in Figs. S2 and S3. The estimated tsunami source of the final model is provided as a separate file (Table S1).


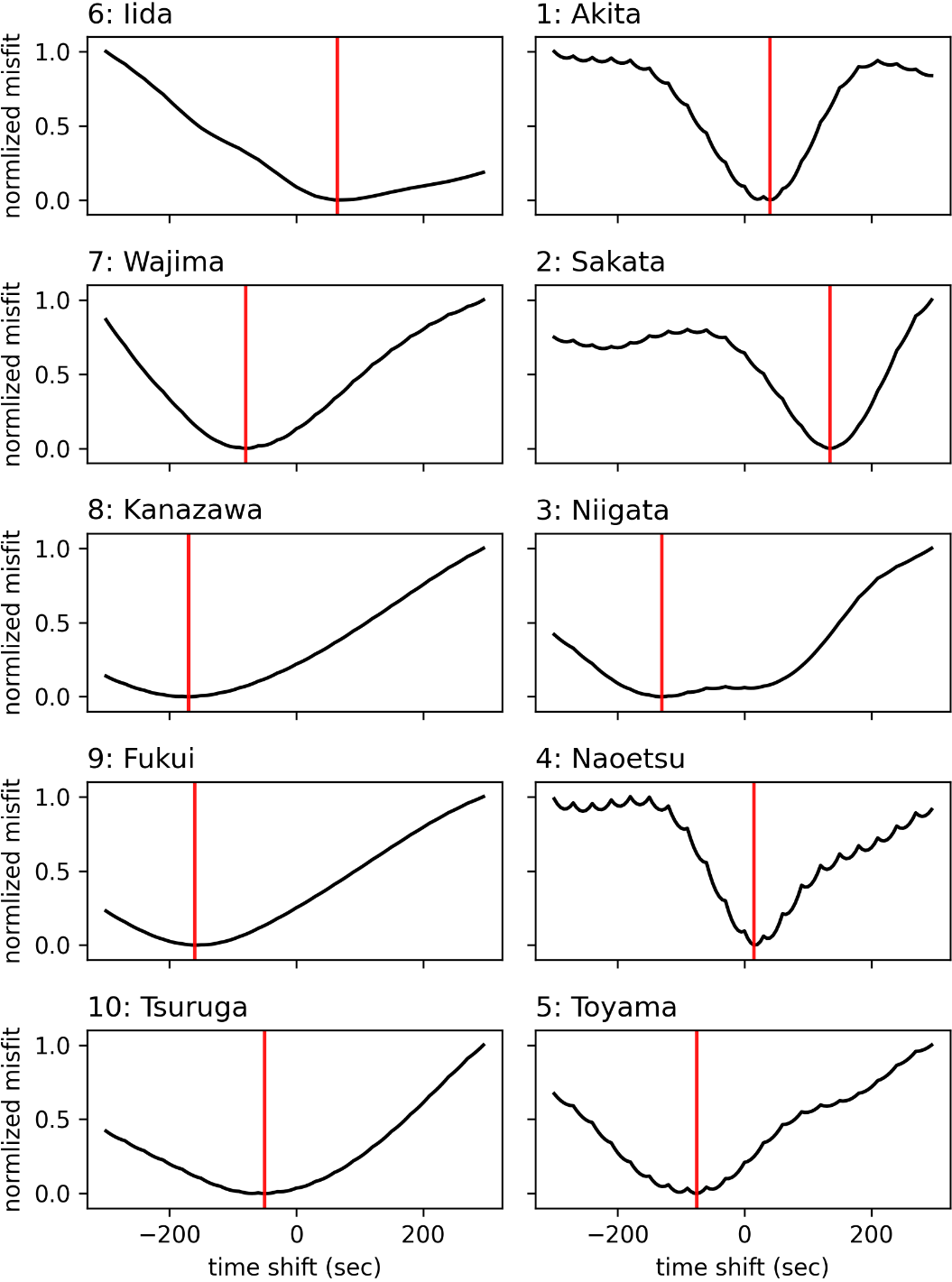


Figure S1. The relationship between time shifts and misfits for each observation point. The misfit at each observation point is defined as the sum of squared differences between the observed waveform and the predicted waveform after applying varying time shifts. Note that each predicted waveform is generated from a tsunami source model estimated without using the data from that particular observation point. The misfit is normalized to range from 0 to 1. The red line indicates the time shift that minimizes the misfit. The plots were created using Matplotlib version 3.10.1 (<https://matplotlib.org/>).


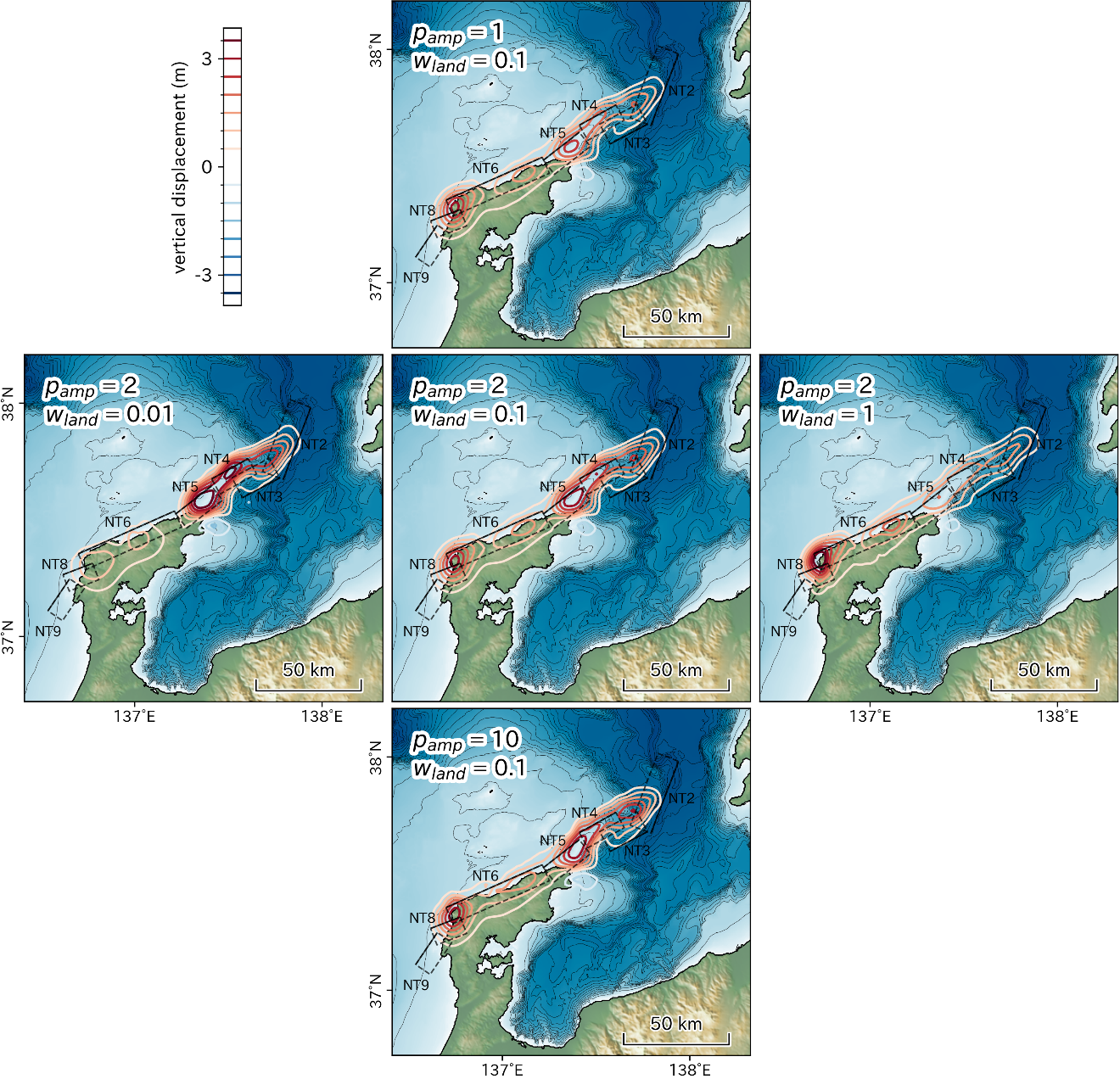


Figure S2. Effect of the two model hyperparameters, $\boldsymbol{p}_{\boldsymbol{amp}}$ and $\boldsymbol{w}_{\boldsymbol{land}}$, on tsunami-source estimation results. The parameter $\boldsymbol{p}_{\boldsymbol{amp}}$ limits the allowable range of the amplitude-error correction coefficient; panels arranged along the vertical axis show the inversion results obtained as $\boldsymbol{p}_{\boldsymbol{amp}}$ is varied. The parameter $\boldsymbol{w}_{\boldsymbol{land}}$ is the weight given to onshore GNSS data; panels arranged along the horizontal axis show the results as $\boldsymbol{w}_{\boldsymbol{land}}$ is varied, with those on the left emphasizing marine observations and those on the right emphasizing terrestrial observations. The central panel corresponds to the results using the selected parameter values. The maps and plots were created using Matplotlib version 3.10.1 (<https://matplotlib.org/>).


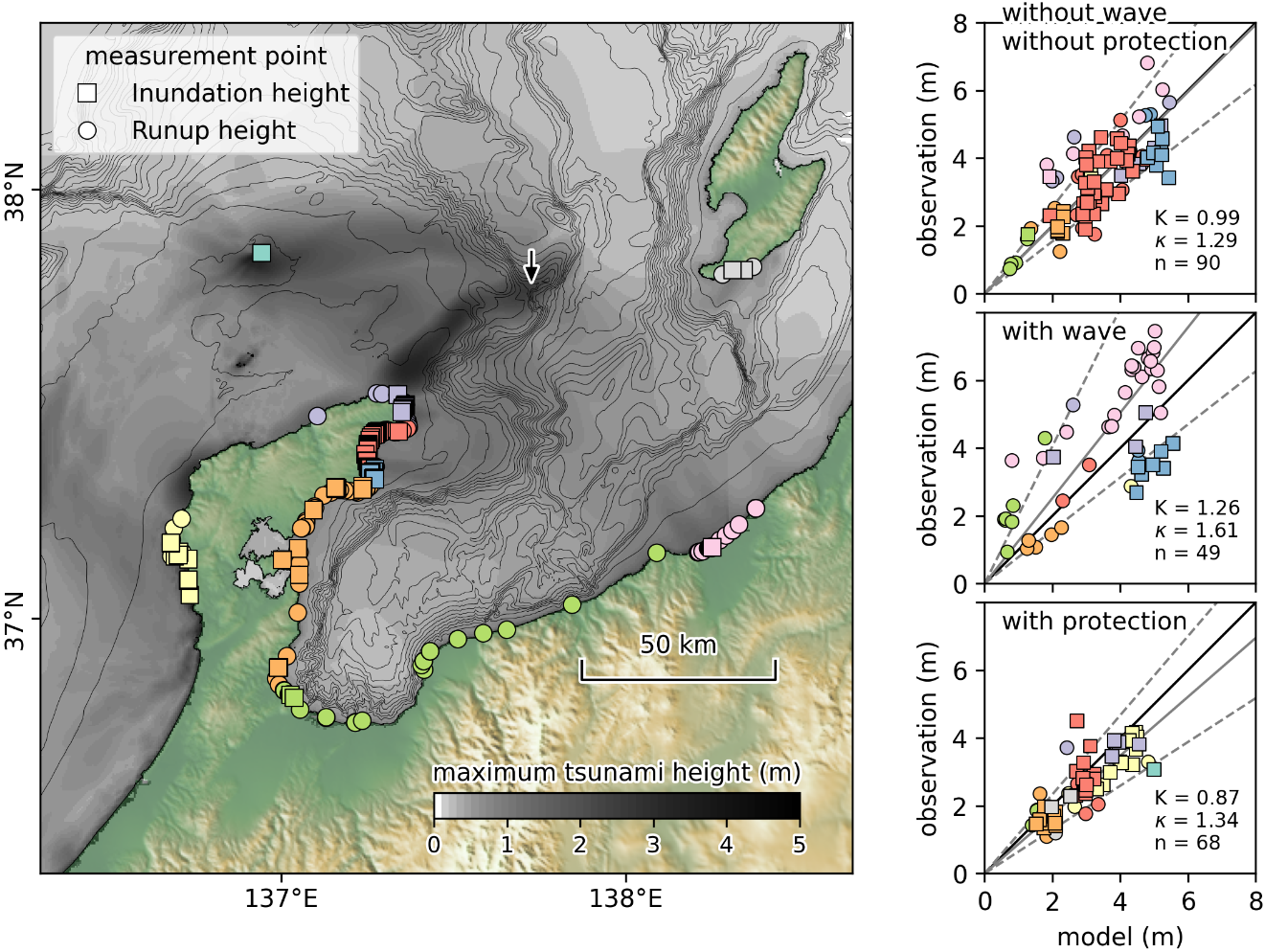
Figure S3. Trace height data (Yuhi et al., 2024b) and model-estimated values. The left panel displays the measurement points for the trace height data, with colors indicating different regions. The arrow marks the location of the uplift peak, estimated near the boundary between the NT2 and NT3 faults. The estimated trace heights from the final model are compared with the measured values in the three panels on the right. The top panel presents data from a site unaffected by waves and unprotected by structures, corresponding to the final model plot in Fig. 2. Note that the vertical and horizontal axes are swapped in this plot. The middle panel shows data from a site affected by waves, while the bottom panel illustrates data from a site protected by structures. Aida's (1978) geometric mean $\boldsymbol{K}$, geometric standard deviation $\boldsymbol{\kappa}$, and the total number of data points $\boldsymbol{n}$ are indicated in each plot. The maps and plots were created using Matplotlib version 3.10.1 (<https://matplotlib.org/>).
